# Frequent Association of Colletotrichum Species with Citrus Fruit and Leaf Spot Disease Symptoms and their Genetic Diversity in Ethiopia

**DOI:** 10.4172/2157-7471.1000425

**Published:** 2017-10-26

**Authors:** Moges AD, Belew D, Admassu B, Yesuf M, Maina S, Ghimire SR

**Affiliations:** 1 Department of Horticulture, Ethiopian Institute of Agricultural Research, Adama, Ethiopia; 2 Department of Horticulture and Plant Sciences, College of Agriculture and Veterinary Medicine, Jimma University, Jimma, Ethiopia; 3 International Institute of Tropical Agriculture (IITA), Nairobi, Kenya; 4 Biosciences Eastern and Central Africa- International Livestock Research Institute (BecA-ILRI) Hub, Nairobi, Kenya; 5 United States Department of Agriculture- The Agricultural Research Service (USDA-ARS), Aberdeen, USA

**Keywords:** DNA barcode marker, Internal transcribed spacer, Long subunit, Actin gene, Pathogenicity, *Colletotrichum*, Citrus

## Abstract

Citrus leaf and fruit spot is one of the most important biotic constraints of citrus production in Ethiopia. The symptomatic leaf and fruit samples were collected from 29 orchards of 15 major citrus growing districts of Ethiopia. One hundred sixty-seven fungal isolates were recovered and identified to species level through DNA barcoding; and their relationships were established using multigene phylogeny. The internal transcribed spacers, long subunit and actin gene sequences revealed that those 167 isolates belonged to either *Collectotrichum gloeosporioides* or *Collectotrichum boninense* species complexes (sensu lato), but no recovery of *Pseudocercospora angolensis*, the primary causal agent of the citrus leaf and fruit spot disease. Detached leaf assays confirmed pathogenicity of isolates of both *C. gloeosporioides* and *C. boninense* species complexes on citrus. They reproduced disease symptoms and the pathogens were re-isolated from symptomatic tissues. This study reports frequent association of *C. gloeosporioides* and *C. boninense* species complexes with citrus fruit and leaf spot disease in Ethiopia. This finding suggests the need for in-depth studies to determine the roles of *C. gloeosporioides* and *C. boninense* species complexes in citrus fruit and leaf spot disease epidemiology.

## Introduction

Citrus (*Citrus* spp.) are economically important fruits in Ethiopia [1,2]. The total acreage and the annual production of citrus are estimated at 7040 hectares and 72459 tons, respectively [3,4]. The commercial citrus farms are located mainly in the central rift valley and the eastern parts of Ethiopia. They contribute about 46% to the total citrus production of the country. Whereas, small-scale citrus productions are scattered throughout the country. Most citrus fruits are consumed fresh while some are processed for juice and marmalade [1]. Sweet oranges and lime are exported to Djibouti, Europe, and the Middle East [5]. However, the citrus production in Ethiopia is severely constrained by diseases including Citrus Fruit and Leaf Spot Disease (CFLSD) [2,6]. The CFLSD is caused by a fungus *Pseudocercospora angolensis* [7]. It can cause yield losses of 20% to 100% [8]. It also affects fruit quality and yields of essential oils [9]. Since the first report in Angola and Mozambique in 1952 [10], the disease has been reported from 22 African countries and Yemen [2,8,11,12]. In Ethiopia, the disease was first reported in 1988 from the southern part of the country [13]. Later, it spread to south, southwest, and northwest parts of Ethiopia and cause heavy crop damage, often total crop loss [2,6].

The CFLSD attacks leaves, fruits, and young twigs [14]. Symptoms on the leaves are characterized by circular, mostly solitary spots with light brown or grayish centers which blacken with sporulation [8]. The lesions are often surrounded by dark brown margins and prominent yellow halos. Infection on fruit produces circular to irregular, discrete or coalescent spots. It also produces yellow halos which is surrounded by tumor-like growth on young fruits [15]. The infection of *P. angolensis* seems to predispose citrus fruits to secondary infection by *Colletotrichum gloeosporioides*. Infection on stem usually occurs from infected petiole. Several lesions on stem may cause defoliation and die back symptoms [10,16]. Despite high economic importance of CFLSD in sub-Saharan Africa, our understanding of biology and epidemiology of the causal agent is very limited [12]. Therefore, this study was conducted to isolate the causal agent associated with symptomatic citrus leaf and fruit samples collected from the major citrus growing areas of Ethiopia and examine pathogen diversity based on the multiple gene sequences and phylogeny.

## Materials and Methods

### Fungal isolates

**Figure 1:**
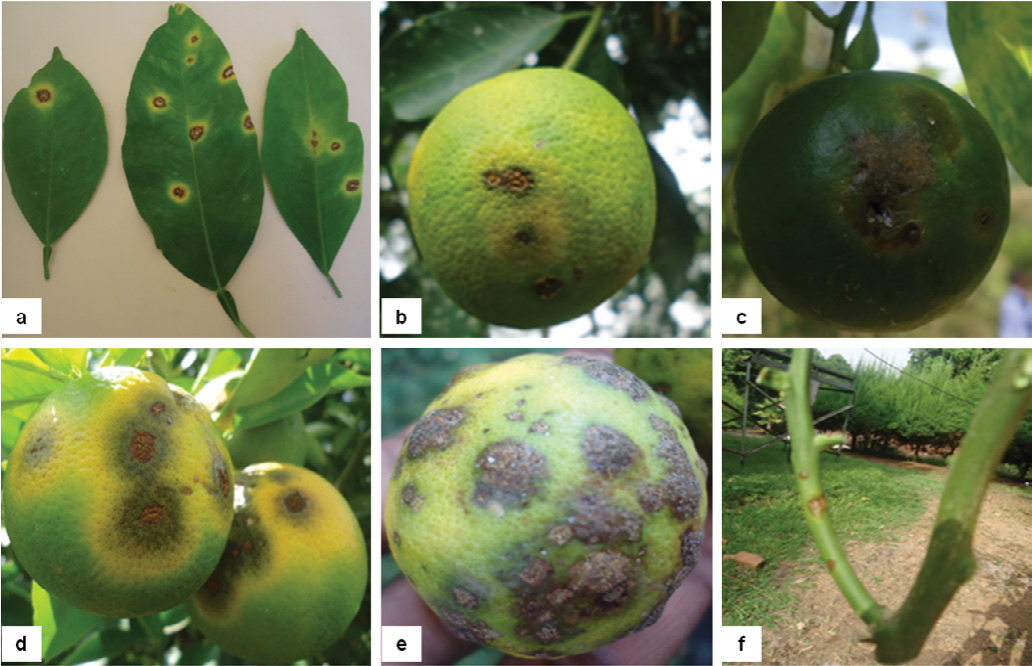
Symptoms of citrus leaf and fruit spot disease in Ethiopia. (a) Leaves with spots, (b to e) symptomatic fruits, and (f) young twig with lesions.

**Figure 2:**
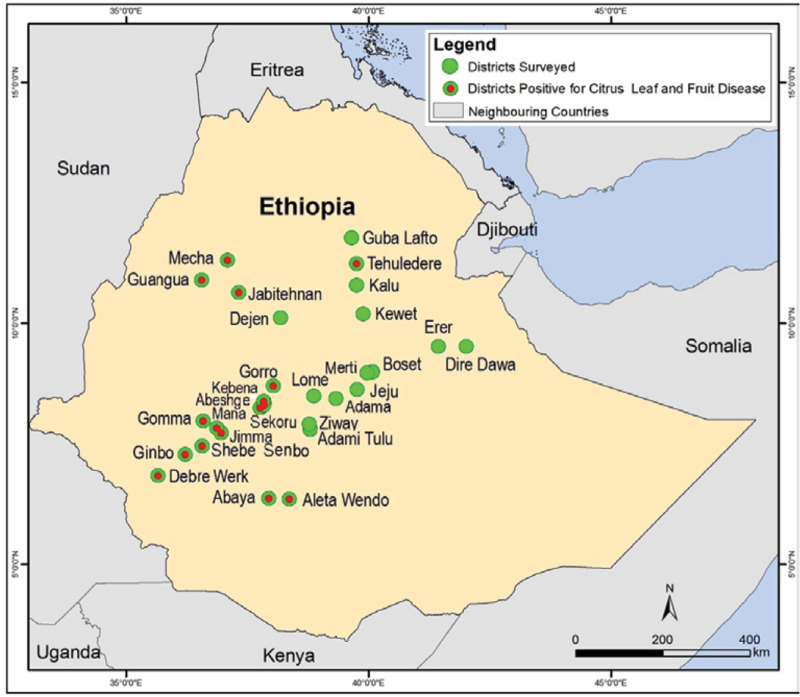
Map of Ethiopia showing the districts surveyed, and the districts with fruit and leaf spot disease symptoms. Locations were plotted as per GPS coordinates using ArcGIS (version 10) software.

Citrus leaves and fruits with distinct symptoms of CFLSD (Figure 1) were collected from citrus production areas of Ethiopia (Figure 2) in between 2012 and 2014. Among 49 citrus orchards surveyed in 29 districts, the disease was recorded in 29 orchards of 15 districts. The symptomatic leaf and fruit samples collected from these orchards were transported to laboratory in ice chest for isolating causal agent. Leaf and fruit tissues comprising both infected and healthy parts were excised, surface disinfected in 70% ethanol for 1 min followed by 1% sodium hypochlorite solution for 10 min and rinsed three times with sterile distilled water. Disinfected tissues were blot dried and placed on potato dextrose agar (PDA; Oxoid, Basingstoke, Hampshire, UK) plates amended with 50 ppm streptomycin sulfate. Cultures were incubated in dark at room temperature. A total of 167 fungi were isolated (Table 1) and monosporic or single-hyphal tip cultures were established. Stock cultures were preserved on PDA slants supplemented with 10% glycerol at 4°C.

**Table 1:** Isolates of Colletotrichum species and their origin in Ethiopia.

Isolate	Host plant	Plant part	Region	District	Collection Site
ETHCTR001	*Citrus sinensis*	Leaf	Southwest Ethiopia	Ginbo	Megenagna
ETHCTR002	*Citrus sinensis*	Fruit	Central Ethiopia	Kebena	Aregita
ETHCTR003	*Citrus sinensis*	Fruit	Central Ethiopia	Geta	Kebul
ETHCTR004	*Citrus sinensis*	Fruit	Central Ethiopia	Abeshege	Rumuga
ETHCTR006	*Citrus sinensis*	Leaf	South Ethiopia	Damot Pulasa	Denba Galie
ETHCTR007	*Citrus sinensis*	Fruit	Central Ethiopia	Abeshege	Holie
ETHCTR008	*Citrus sinensis*	Leaf	Southwest Ethiopia	Debre Werk	Bebeka
ETHCTR009	*Citrus sinensis*	Leaf	Southwest Ethiopia	Mana	Gube Bosoka
ETHCTR012	*Citrus sinensis*	Leaf	Southwest Ethiopia	Shebe Senbo	Kishe-Kosta
ETHCTR013	*Citrus sinensis*	Leaf	Central Ethiopia	Abeshege	Jejeba
ETHCTR014	*Citrus sinensis*	Leaf	Central Ethiopia	Abeshege	Jejeba
ETHCTR016	*Citrus aurantium*	Leaf	Central Ethiopia	Wolisso	Fodu Gora
ETHCTR017	*Citrus aurantium*	Fruit	Central Ethiopia	Wolisso	Fodu Gora
ETHCTR018	*Citrus sinensis*	Leaf	Central Ethiopia	Wolisso	Fodu Gora
ETHCTR019	*Citrus aurantium*	Leaf	Central Ethiopia	Wolisso	Fodu Gora
ETHCTR020	*Citrus sinensis*	Leaf	Central Ethiopia	Abeshege	Holie
ETHCTR021	*Citrus sinensis*	Leaf	South Ethiopia	Damot Pulasa	Denba Galie
ETHCTR022	*Citrus sinensis*	Leaf	South Ethiopia	Damot Pulasa	Denba Galie
ETHCTR023	*Citrus sinensis*	Leaf	South Ethiopia	Damot Pulasa	Denba Galie
ETHCTR024	*Citrus sinensis*	Leaf	Central Ethiopia	Cheha	Chifangira
ETHCTR025	*Citrus sinensis*	Leaf	Central Ethiopia	Cheha	Chifangira
ETHCTR026	*Citrus sinensis*	Leaf	Central Ethiopia	Cheha	Sisena Mitia
ETHCTR027	*Citrus sinensis*	Leaf	Central Ethiopia	Cheha	Sisena Mitia
ETHCTR028	*Citrus sinensis*	Leaf	Central Ethiopia	Cheha	Sisena Mitia
ETHCTR029	*Citrus sinensis*	Leaf	Central Ethiopia	Cheha	Sisena Mitia
ETHCTR031	*Citrus sinensis*	Leaf	South Ethiopia	Damot Pulasa	Denba Galie
ETHCTR032	*Citrus sinensis*	Leaf	South Ethiopia	Boloso Sore	Areka
ETHCTR033	*Citrus sinensis*	Fruit	South Ethiopia	Boloso Sore	Areka
ETHCTR034	*Citrus sinensis*	Fruit	South Ethiopia	Boloso Sore	Areka
ETHCTR037	*Citrus sinensis*	Fruit	Central Ethiopia	Sekoru	Gibe
ETHCTR038	*Citrus reticulate*	Leaf	Central Ethiopia	Sekoru	Gibe
ETHCTR039	*Citrus sinensis*	Leaf	South Ethiopia	Abaya	Guangua
ETHCTR041	*Citrus reticulate*	Leaf	Northwest Ethiopia	Jabitehnan	Finoteselam
ETHCTR042	*Citrus sinensis*	Leaf	Northwest Ethiopia	Jabitehnan	Finoteselam
ETHCTR043	*Citrus sinensis*	Fruit	Northwest Ethiopia	Jabitehnan	Finoteselam
ETHCTR044	*Citrus sinensis*	Fruit	Northwest Ethiopia	Jabitehnan	Finoteselam
ETHCTR045	*Citrus sinensis*	Leaf	Southwest Ethiopia	Mana	Gube Bosoka
ETHCTR046	*Citrus sinensis*	Fruit	Southwest Ethiopia	Mana	Gube Bosoka
ETHCTR047	*Citrus sinensis*	Leaf	Central Ethiopia	Gorro	Adami Wedessa
ETHCTR048	*Citrus aurantium*	Leaf	South Ethiopia	Abaya	Guangua
ETHCTR049	*Citrus sinensis*	Leaf	Southwest Ethiopia	Gomma	Agaro
ETHCTR050	*Citrus aurantium*	Leaf	South Ethiopia	Abaya	Lado
ETHCTR051	*Citrus sinensis*	Fruit	South Ethiopia	Abaya	Guangua
ETHCTR052	*Citrus sinensis*	Leaf	Southwest Ethiopia	Gomma	Agaro
ETHCTR053	*Citrus sinensis*	Leaf	Central Ethiopia	Abeshege	Gasorie
ETHCTR054	*Citrus sinensis*	Fruit	Southwest Ethiopia	Gomma	Agaro
ETHCTR055	*Citrus sinensis*	Leaf	Southwest Ethiopia	Mana	Gube Bosoka
ETHCTR056	*Citrus sinensis*	Leaf	South Ethiopia	Abaya	Guangua
ETHCTR057	*Citrus sinensis*	Leaf	South Ethiopia	Abaya	Guangua
ETHCTR058	*Citrus sinensis*	Fruit	Central Ethiopia	Abeshege	Gasorie
ETHCTR059	*Citrus sinensis*	Leaf	Northwest Ethiopia	Guangua	Chagni
ETHCTR060	*Citrus sinensis*	Leaf	Northwest Ethiopia	Guangua	Chagni
ETHCTR061	*Citrus sinensis*	Fruit	Northwest Ethiopia	Jabitehnan	Finoteselam
ETHCTR062	*Citrus sinensis*	Fruit	Northwest Ethiopia	Jabitehnan	Finoteselam
ETHCTR063	*Citrus sinensis*	Leaf	South Ethiopia	Abaya	Guangua
ETHCTR064	*Citrus sinensis*	Fruit	South Ethiopia	Abaya	Guangua
ETHCTR065	*Citrus sinensis*	Fruit	Central Ethiopia	Kebena	Aregita
ETHCTR066	*Citrus sinensis*	Fruit	Central Ethiopia	Kebena	Aregita
ETHCTR067	*Citrus sinensis*	Fruit	Southwest Ethiopia	Debre Werk	Bebeka
ETHCTR068	*Citrus sinensis*	Leaf	Southwest Ethiopia	Debre Werk	Bebeka
ETHCTR069	*Citrus sinensis*	Leaf	Southwest Ethiopia	Debre Werk	Bebeka
ETHCTR070	*Citrus sinensis*	Leaf	Central Ethiopia	Kebena	Aregita
ETHCTR071	*Citrus sinensis*	Leaf	Central Ethiopia	Kebena	Aregita
ETHCTR072	*Citrus reticulate*	Fruit	Northwest Ethiopia	Jabitehnan	Finoteselam
ETHCTR073	*Citrus reticulate*	Fruit	Northwest Ethiopia	Jabitehnan	Finoteselam
ETHCTR074	*Citrus sinensis*	Leaf	South Ethiopia	Aleta Wendo	Omacho Chawa
ETHCTR075	*Citrus sinensis*	Leaf	South Ethiopia	Aleta Wendo	Omacho Chawa
ETHCTR077	*Citrus sinensis*	Leaf	Northwest Ethiopia	Guangua	Chagni
ETHCTR078	*Citrus sinensis*	Fruit	Central Ethiopia	Abeshege	Tawela
ETHCTR081	*Citrus sinensis*	Fruit	Central Ethiopia	Abeshege	Gasorie
ETHCTR082	*Citrus sinensis*	Fruit	Central Ethiopia	Abeshege	Gasorie
ETHCTR084	*Citrus sinensis*	Leaf	Southwest Ethiopia	Ginbo	Megenagna
ETHCTR085	*Citrus sinensis*	Leaf	Southwest Ethiopia	Ginbo	Megenagna
ETHCTR086	*Citrus sinensis*	Leaf	Southwest Ethiopia	Shebe Senbo	Kishe-Kosta
ETHCTR088	*Citrus sinensis*	Leaf	Northwest Ethiopia	Jabitehnan	Finoteselam
ETHCTR089	*Citrus sinensis*	Fruit	Southwest Ethiopia	Mana	Gube Bosoka
ETHCTR090	*Citrus sinensis*	Leaf	Northwest Ethiopia	Jabitehnan	Finoteselam
ETHCTR091	*Citrus sinensis*	Leaf	Northwest Ethiopia	Jabitehnan	Finoteselam
ETHCTR092	*Citrus sinensis*	Fruit	Northwest Ethiopia	Guangua	Chagni
ETHCTR093	*Citrus sinensis*	Fruit	Northwest Ethiopia	Guangua	Chagni
ETHCTR094	*Citrus sinensis*	Leaf	South Ethiopia	Abaya	Guangua
ETHCTR095	*Citrus sinensis*	Leaf	Northwest Ethiopia	Guangua	Chagni
ETHCTR096	*Citrus sinensis*	Leaf	Southwest Ethiopia	Shebe Senbo	Kishe-Kosta
ETHCTR097	*Citrus sinensis*	Fruit	South Ethiopia	Abaya	Guangua
ETHCTR098	*Citrus sinensis*	Leaf	South Ethiopia	Boloso Sore	Areka
ETHCTR101	*Citrus sinensis*	Leaf	Central Ethiopia	Abeshege	Layignaw Tatesa
ETHCTR103	*Citrus aurantium*	Leaf	South Ethiopia	Abaya	Lado
ETHCTR104	*Citrus sinensis*	Leaf	Southwest Ethiopia	Gomma	Agaro
ETHCTR105	*Citrus sinensis*	Leaf	Southwest Ethiopia	Gomma	Agaro
ETHCTR106	*Citrus sinensis*	Leaf	Southwest Ethiopia	Gomma	Agaro
ETHCTR107	*Citrus sinensis*	Fruit	Southwest Ethiopia	Gomma	Agaro
ETHCTR108	*Citrus sinensis*	Leaf	Northwest Ethiopia	Jabitehnan	Finoteselam
ETHCTR109	*Citrus sinensis*	Leaf	Northwest Ethiopia	Jabitehnan	Finoteselam
ETHCTR110	*Citrus sinensis*	Leaf	Northwest Ethiopia	Jabitehnan	Finoteselam
ETHCTR111	*Citrus sinensis*	Fruit	Northwest Ethiopia	Jabitehnan	Finoteselam
ETHCTR112	*Citrus sinensis*	Leaf	Central Ethiopia	Abeshege	Layignaw Tatesa
ETHCTR113	*Citrus sinensis*	Leaf	Central Ethiopia	Abeshege	Layignaw Tatesa
ETHCTR114	*Citrus sinensis*	Leaf	North Central	Tehuledere	Hayk/Jarre
ETHCTR115	*Citrus sinensis*	Leaf	South Ethiopia	Aleta Wendo	Omacho Chawa
ETHCTR116	*Citrus sinensis*	Leaf	South Ethiopia	Aleta Wendo	Omacho Chawa
ETHCTR117	*Citrus sinensis*	Leaf	Southwest Ethiopia	Mana	Gube Bosoka
ETHCTR118	*Citrus reticulate*	Fruit	Northwest Ethiopia	Jabitehnan	Finoteselam
ETHCTR119	*Citrus reticulate*	Fruit	Northwest Ethiopia	Jabitehnan	Finoteselam
ETHCTR120	*Citrus reticulate*	Fruit	Northwest Ethiopia	Jabitehnan	Finoteselam
ETHCTR121	*Citrus reticulate*	Fruit	Northwest Ethiopia	Jabitehnan	Finoteselam
ETHCTR122	*Citrus sinensis*	Leaf	Southwest Ethiopia	Ginbo	Balewold
ETHCTR123	*Citrus sinensis*	Leaf	Southwest Ethiopia	Ginbo	Balewold
ETHCTR124	*Citrus sinensis*	Leaf	Southwest Ethiopia	Ginbo	Balewold
ETHCTR127	*Citrus sinensis*	Leaf	Southwest Ethiopia	Shebe Senbo	Kishe-Kosta
ETHCTR128	*Citrus sinensis*	Leaf	Southwest Ethiopia	Shebe Senbo	Kishe-Kosta
ETHCTR129	*Citrus sinensis*	Leaf	Southwest Ethiopia	Debre Werk	Bebeka
ETHCTR130	*Citrus sinensis*	Leaf	Southwest Ethiopia	Debre Werk	Bebeka
ETHCTR131	*Citrus sinensis*	Leaf	Southwest Ethiopia	Debre Werk	Bebeka
ETHCTR132	*Citrus sinensis*	Fruit	Central Ethiopia	Abeshege	Tawela
ETHCTR133	*Citrus sinensis*	Fruit	Central Ethiopia	Abeshege	Tawela
ETHCTR134	*Citrus sinensis*	Leaf	Central Ethiopia	Abeshege	Gasorie
ETHCTR136	*Citrus sinensis*	Leaf	Central Ethiopia	Abeshege	Gasorie
ETHCTR137	*Citrus sinensis*	Fruit	Central Ethiopia	Abeshege	Gasorie
ETHCTR138	*Citrus sinensis*	Fruit	Central Ethiopia	Abeshege	Gasorie
ETHCTR139	*Citrus sinensis*	Leaf	Southwest Ethiopia	Debre Werk	Bebeka
ETHCTR140	*Citrus sinensis*	Leaf	Southwest Ethiopia	Debre Werk	Bebeka
ETHCTR141	*Citrus sinensis*	Leaf	Southwest Ethiopia	Debre Werk	Bebeka
ETHCTR142	*Citrus sinensis*	Leaf	Southwest Ethiopia	Debre Werk	Bebeka
ETHCTR143	*Citrus sinensis*	Leaf	Southwest Ethiopia	Debre Werk	Bebeka
ETHCTR146	*Citrus sinensis*	Leaf	Southwest Ethiopia	Gomma	Agaro
ETHCTR148	*Citrus sinensis*	Leaf	Central Ethiopia	Abeshege	Gasorie
ETHCTR151	*Citrus sinensis*	Fruit	Southwest Ethiopia	Debre Werk	Bebeka
ETHCTR152	*Citrus sinensis*	Fruit	Southwest Ethiopia	Debre Werk	Bebeka
ETHCTR153	*Citrus sinensis*	Fruit	Southwest Ethiopia	Debre Werk	Bebeka
ETHCTR154	*Citrus sinensis*	Fruit	Northwest Ethiopia	Jabitehnan	Finoteselam
ETHCTR156	*Citrus sinensis*	Fruit	South Ethiopia	Abaya	Guangua
ETHCTR157	*Citrus sinensis*	Leaf	Southwest Ethiopia	Debre Werk	Bebeka
ETHCTR158	*Citrus sinensis*	Leaf	Southwest Ethiopia	Debre Werk	Bebeka
ETHCTR159	*Citrus sinensis*	Leaf	Southwest Ethiopia	Debre Werk	Bebeka
ETHCTR160	*Citrus sinensis*	Leaf	Southwest Ethiopia	Gomma	Agaro
ETHCTR161	*Citrus sinensis*	Leaf	Northwest Ethiopia	Guangua	Chagni
ETHCTR162	*Citrus sinensis*	Fruit	Northwest Ethiopia	Jabitehnan	Finoteselam
ETHCTR163	*Citrus sinensis*	Leaf	Northwest Ethiopia	Jabitehnan	Finoteselam
ETHCTR164	*Citrus sinensis*	Leaf	Northwest Ethiopia	Jabitehnan	Finoteselam
ETHCTR165	*Citrus sinensis*	Leaf	Northwest Ethiopia	Jabitehnan	Finoteselam
ETHCTR166	*Citrus sinensis*	Fruit	South Ethiopia	Boloso Sore	Areka
ETHCTR167	*Citrus sinensis*	Fruit	South Ethiopia	Boloso Sore	Areka
ETHCTR169	*Citrus sinensis*	Fruit	South Ethiopia	Boloso Sore	Areka
ETHCTR170	*Citrus sinensis*	Leaf	Southwest Ethiopia	Ginbo	Megenagna
ETHCTR172	*Citrus sinensis*	Leaf	Southwest Ethiopia	Ginbo	Megenagna
ETHCTR173	*Citrus sinensis*	Leaf	Southwest Ethiopia	Mana	Gube Bosoka
ETHCTR174	*Citrus sinensis*	Leaf	Southwest Ethiopia	Mana	Gube Bosoka
ETHCTR175	*Citrus reticulata*	Leaf	Northwest Ethiopia	Jabitehnan	Finoteselam
ETHCTR176	*Citrus reticulata*	Leaf	Northwest Ethiopia	Jabitehnan	Finoteselam
ETHCTR178	*Citrus reticulata*	Leaf	Northwest Ethiopia	Jabitehnan	Finoteselam
ETHCTR179	*Citrus reticulata*	Leaf	Northwest Ethiopia	Jabitehnan	Finoteselam
ETHCTR180	*Citrus reticulata*	Leaf	Northwest Ethiopia	Jabitehnan	Finoteselam
ETHCTR181	*Citrus sinensis*	Fruit	Northwest Ethiopia	Jabitehnan	Finoteselam
ETHCTR182	*Citrus sinensis*	Fruit	Northwest Ethiopia	Jabitehnan	Finoteselam
ETHCTR183	*Citrus sinensis*	Fruit	Northwest Ethiopia	Jabitehnan	Finoteselam
ETHCTR184	*Citrus sinensis*	Fruit	Northwest Ethiopia	Jabitehnan	Finoteselam
ETHCTR185	*Citrus sinensis*	Fruit	Northwest Ethiopia	Jabitehnan	Finoteselam
ETHCTR186	*Citrus sinensis*	Fruit	Northwest Ethiopia	Jabitehnan	Finoteselam
ETHCTR187	*Citrus sinensis*	Leaf	Central Ethiopia	Cheha	Sisena Mitia
ETHCTR188	*Citrus sinensis*	Fruit	Central Ethiopia	Geta	Kebul
ETHCTR189	*Citrus sinensis*	Leaf	South Ethiopia	Damot Pulasa	Denba Galie
ETHCTR190	*Citrus sinensis*	Leaf	South Ethiopia	Damot Pulasa	Denba Galie
ETHCTR192	*Citrus sinensis*	Leaf	Northwest Ethiopia	Jabitehnan	Finoteselam
ETHCTR193	*Citrus sinensis*	Fruit	Northwest Ethiopia	Jabitehnan	Finoteselam
ETHCTR194	*Citrus sinensis*	Leaf	Central Ethiopia	Abeshege	Holie
ETHCTR197	*Citrus aurantium*	Leaf	Central Ethiopia	Wolisso	Fodu Gora
ETHCTR198	*Citrus aurantium*	Fruit	Central Ethiopia	Wolisso	Fodu Gora

### DNA extraction

Fungal genomic DNA was extracted using the procedures described earlier [17,18] with some modifications. Mycelium was scraped from the surface of a week-old cultures, added with fine sand, and crushed in a mortar and pestle. The finely ground mycelium was transferred into 1.5 ml Eppendorf tubes with sterile glass beads, and were pulverized with GenoGrinder at 25 rpm for 3 min in 0.5 ml extraction buffer (3% CTAB, 200 mM Tris-HCl (pH 8.0), 0.5 M NaCl, 10 mM EDTA (pH 8.0), and 2% SDS (preheated at 65°C)). To each sample, 150 μl of sodium acetate (3 M, pH 5.2) was added and mixed by inverting the tube. The mixtures were incubated at -20°C for 10 min and centrifuged at 14800 rpm for 10 min at room temperature. The supernatant was transferred into a new Eppendorf tube and 300 μl of ice-cold isopropanol was added and mixed by gentle inverting. The mixture was kept at room temperature for 5 min. Precipitated DNA was pelleted by centrifugation. The supernatant was poured off and the DNA pellet was washed with 0.5 ml of 70% ethanol and centrifuged for 5 min. The supernatant was decanted, and the pellet was dried at room temperature by placing the tubes face down on paper towels for 20 min. The pellet was re-suspended in 50 μl low-salt TE buffer and stored at -20°C. The concentration and quality of DNA was checked by NanoDrop 2000c Spectrophotometer (Thermo Fisher Scientific, Walthum, MA, USA), and visualized on a 1% agarose gel (Sigma-Aldrich, Saint Louis, MO, USA) stained with SYBR Safe DNA gel stain under ultra-violet light (UVP BioImaging Systems, Upland, CA, USA).

### PCR amplification

Three loci including the 5.8 S nuclear ribosomal gene with the two flanking ITS regions, the portion of Large Sub Unit (LSU), and partial sequences of the ACT gene were amplified and sequenced using universal primer pairs ITS-1F/ITS-4 [19,20], LROR/LR5 [21,22], and ACT-512F/ACT-783R [23], respectively. Genomic DNA from *Acremonium* species isolate 133 was used as positive control whereas reaction with no DNA template was used for negative control.

All PCRs were performed in 20 µl reaction volumes containing AccuPower PCR PreMix (Bioneer, Daejeon, Republic of Korea), 0.8 μl of 10 µM of each forward and reverse primers, and 2 µl template DNA (20 ng/μl). PCR reactions were performed in a GeneAmp PCR System 9700 thermal cycler (Applied Biosystems, Foster City, CA, USA). The PCR conditions for the three loci were optimized using gradient PCR. The PCR cycling conditions for the ITS regions constituted an initial denaturation step at 94°C for 5 min, followed by 35 cycles of denaturation at 94°C for 45 secs, primer annealing at 48°C for 45 sec, and primer extension at 72°C for 1 min; and a final extension step at 72°C for 10 min. The PCR programs for LSU were an initial denaturation step at 95°C for 5 min, followed by 35 cycles at 94°C for 30 sec, at 43°C for 30 sec, and at 72°C for 1 min; and a final extension step at 72°C for 10 min. PCR reaction profiles for partial ACT gene comprised an initial denaturation at 96°C for 2 min, followed by 35 cycles at 94°C for 30 sec, at 61°C for 45 sec, and at 72°C for 45 sec; and a final extension step at 72°C for 10 min.

PCR products were subjected to electrophoresis in 1.5% agarose gels stained with SYBR Safe DNA gel stain at 70 V for 45 min and visualized under UV light (UVP BioImaging Systems). The sizes of amplicons were determined against a 100 bp molecular weight marker (Invitrogen, Carlsbad, CA, USA). The PCR products with the expected sizes were cleaned using the GeneJET (Thermo Fisher Scientific) for ITS and QIAquick (Qiagen, Venlo, The Netherlands) PCR purification kit for LSU and ACT as instructed by the manufacturers. The concentration and quality of the purified PCR products were determined by NanoDrop 2000c spectrophotometer (Thermo Fisher Scientific) and visualized on 1.5% agarose gel electrophoresis.

### DNA sequencing and alignment

Purified PCR products were sequenced using the same forward and reverse primers used for PCR amplifications with BigDye Terminator v3.1 Cycle Sequencing Kit (Applied Biosystems) and were run on an ABI 3130x l DNA analyzer (Applied Biosystems) at BecA-ILRI Hub, Nairobi, Kenya.

The nucleotide sequence datasets obtained from forward and reverse primers were inspected, edited, and assembled into consensus contigs using CLC Main Workbench v7.5.1 (CLC bio, Prismet, Denmark). The sequences were analyzed using BLASTN v2.2.30 (http://blast.ncbi.nlm.nih.gov/Blast.cgi) program [24] against the GenBank database based on the best hits of the query sequences that were used to assign identities to the test isolates. Multiple sequence alignments were performed with MAFFT v7.221 [25] using the auto alignment strategy with the 200 PAM/ K=2 scoring matrix and a gap opening penalty of 1.53 with an offset value of 0.0. The ambiguous regions of each gene sequences were removed with Gblocks v0.91b [26]. Resulting sequence alignments were evaluated and manually edited where necessary using MEGA v6.06 [27] software.

### Phylogenetic analyses

Phylogenetic analyses were performed for each multiple sequence alignment of the ITS, LSU, and ACT as well as for the combined dataset of the three loci using different statistical methods to differentiate the isolates by species complex. jModelTest v2.1.7 [28] as well as the Modeltest [29] implemented in the MEGA were used to estimate the best-fit models of nucleotide substitution and the corresponding general time-reversible (GTR) substitution rate parameters, shape of the four-category gamma distribution and fraction of invariable sites for each gene using corrected Akaike Information Criterion (AICc) and the Bayesian Information Criterion (BIC) scores. For each locus, 167 sequence datasets were used to reconstruct phylogenetic trees. Published ITS, LSU and ACT nucleotide sequences of 13 isolates of *Colletotrichum* spp. from the GenBank database were included as reference species (Table 2). *Colletotrichum acutatum* J. H. Simmonds (GenBank accession numbers: DQ286124, DQ286125, and JQ949687) [30,31] was designated as outgroup in all analyses for the reconstruction of the phylogenetic trees.

**Table 2:** Details of reference isolates used in this study.

Species	Accession number^a^	Host	Country	GenBank number^b^			Reference
				ITS	LSU	ACT	
*C. aenigma*	ICMP 18608, LC0038, C1253.4	*Persea americana*	Israel	NR_120140	JN940409	JX009443	Cai and Weir [41]
*C. aotearoa*	C1252.9, AR2802, ICMP 18532	Kunzea ericoides, Pueraria, Vitexlucens	New Zealand, USA	JX010198	DQ286187	JX009544	Farr et al. [31]; Weir et al. [41]
*C. asianum*	C1187, LC0036, CPC 20981	*Mangifera indica*, unknown, Fruits	Australia, Thailand, Brazil	JX010192	JN940407	KC566879	Cai 2011; Weir et al. [41]; Braganca (unpublished data)
*C. boninense*	CBS 128547, ICMP 10338	*Camellia* sp.	New Zeealand	JQ005159	DQ286169	JQ005507	Farr et al. [31]; Damm et al.
*C. fructicola*	ICMP 12568, LC0032, CMM3811	Persea americana, unknown, Mangiferaindica	Australia, Thailand, Brazil	JX010166	JN940418	KC702919	Cai 2011; Weir et al. [41]; Chowdappa and Chethana2014 (unpublished data)
*C. gloeosporioides*	OCAC24, LC0553, C1254.3	Elettariacardamomum, unknown, Citrus sp.	India, China, USA	KJ813602	JN940414	JX009494	Cai 2011; Weir et al. [41]; Chowdappa and Chethana2014 (unpublished data)
*C. gloeosporioides*	GM62-L03, PP143, CPC 20904	*Annona muricata*; Fruits	Colombia, Brazil	KC512137	FJ890371	KC566853	Gazis and Chaverri (unpublished data); Braganca (unpublished data); Alvarezet al.
*C. gloeosporioides*	Strain 8, GJS01-199, CBS 953.97	Olive, *Citrus sinensis*, Theobroma	Italy, Cameroon	JN121209	DQ286177	GQ856782	Farr et al. [31]; Yang et al. (unpublished data); Faeddaet al.
*C. gloeosporioides*	CK13b7, AR4031, CBS 131329	*Citrus limon*, Fruits	Cameroon, Brazil	JX436791	AY539807	KC566856	Berner et al. (unpublished data); Braganca (unpublished data); Douanla-Meli et al. (unpublished data)
C. gloeosporioides	M2P3D7, CBS 122687, C1014.6	Soybean, Leucospermum sp., Citrus sp.,	Brazil, SouthAfrica, NewZealand	JX258787	EU552111	JX009462	Marincowitz et al. unpublished data); Cnossen-Fassoni et al. (unpublisheddata); Weir et al.
C. karstii	OCAC4, CBS 102667, BRIP:28443a	Elettariacardamomum; Passiflora; Mangifera indica	India, NewZealand, Australia	KJ813595	DQ286173	JQ005551	Farr et al., Damm et al.; Chowdappa and Chethana (unpublished data)
C. siamense	ICMP 12567, FAU 553, CBS 114054/BPI 747978 C1315.2	Persea americana, Fragaria, Coffeaarabica	Australia, USA, Thailand	JX010250	AF543786	JX009518	Farr et al., Damm et al.; Chowdappa and Chethana (unpublished data)
Glomerella cingulata	ICMP 10646, AR 2799	Camellia sasanqua, Pueraria lobata	USA	JX010225	DQ286193	JX009563	Farra et al.; Weir et al. 2012
C. acutatum (outgroup)	MEP1323, CBS:126521	Vaccinium, Anemone F1 hybrid	New Zealand, Netherlands	DQ286124	DQ286125	JQ949687	Farr et al. and Damm et al.

^a^ BPI: The U.S. National Fungus Collections; BRIP: Plant Pathology Herbarium, Department of Primary Industries, Queensland, Australia; CBS: Culture collection of the Centraalbureau voor Schimmelcultures, Fungal Biodiversity Centre, Utrecht, The Netherlands; CMM: Culture Collection of Phytopathogenic Fungi Prof. Maria Menezes (Colecao de Culturas de Fungos Fitopatogenicos Prof. Maria Menezes), Brazil; CPC: Culture collection of Pedro Crous, housed at CBS; FAU: Florida Atlantic University, Harbor Branch Marine Microbial Database, Boca Raton, Florida; ICMP: International Collection of Microorganisms from Plants; GJS: Gary J. Samuels searchable database;

^b^ ITS: internal transcribed spacers and intervening 5.8S nrDNA; LSU: partial long subunit of nrDNA gene; ACT: partial actin gene.

To determine whether the three sequence datasets were congruent and combinable, tree topologies of 70% reciprocal Neighbor-joining (NJ) bootstrap with Maximum Likelihood (ML) distances (1000 replicates) with substitution models determined separately for each partition using Model test were compared visually [32]. The analyses showed that individual genes were broadly congruent, thus nucleotide alignments of the three genes were concatenated using scripts in Microsoft Office Excel 2007 program. Phylogeny reconstruction was performed by NJ method [33] using the MEGA software. The percentage of replicate trees in which the associated taxa clustered together was evaluated with a bootstrap analysis with 1000 replicates [34]. The tree was drawn to scale, with branch lengths in the same units as those of the evolutionary distances used to infer the phylogenetic tree. The evolutionary distances were computed using the Kimura 2- parameter substitution model [35]. All alignment positions containing gaps and missing data were removed and the rate variation among sites was modeled with a gamma distribution (shape parameter=5). All branches with bootstrap values of less than 50 were collapsed.

The evolutionary history was inferred using the Maximum Parsimony (MP) method on the combined multilocus alignments using Tree-Bisection-Reconstruction (TBR) algorithm with search level 3 in which the initial trees were obtained by 10 random sequence additions. Alignment gaps and missing data were eliminated and the rate of variation among sites was modeled with gamma distribution. The confidence values for clades within the resulting tree were determined using a bootstrap analysis with 1000 replicates [34]. Tree length (TL), consistency index (CI), retention index (RI), rescaled consistency index (RC) and homoplasy index (HI) were calculated for one of the most parsimony trees.

A Markov Chain Monte Carlo (MCMC) algorithm was used to generate phylogenetic trees with Bayesian probabilities using MrBayes v3.2.1 [36] for the combined multilocus sequence datasets. Based on the results of the jModelTest, the Bayesian analysis for all loci was performed using the Dirichlet (1,1,1,1) nucleotide frequency distribution, and GTR model with gamma-distributed rate variation across sites and a proportion of invariable sites. The analyses of two MCMC chains on the full data set were run from random trees for 4 × 10^6^ generations and sampled from the posterior every 1000 generations until the split frequency reached below 0.02. The first 25% of the trees were discarded as burn-in phase of the analysis and posterior probabilities were determined from the remaining trees. The effective sample size and traces of all parameters and convergence of the two runs were checked using the internal diagnostics of the standard deviation of split frequencies and performance scale reduction factors, and then checked externally with Tracer v1.6 [37]. A summary maximum clade credibility species tree was built with TreeAnnotator v1.7.1 [38] using a 25% burn-in and a posterior probability limit of 0.5. The resulting phylogenetic trees were drawn and edited using TreeGraph v2.4.0-456 beta [39]. All tree branches with values of less than 0.50 were collapsed.

Sequences derived in the present study were deposited in the GenBank with accession numbers from KT282463 to KT282629 (ACT), from KT282630 to KT282796 (ITS), and from KT282797 to KT282963 (LSU), whereas alignments and trees were deposited into TreeBASE (http://purl.org/phylo/treebase/phylows/study/TB2:S17920).

### Pathogenicity tests

Representative isolates of *C. gloeosporioides* and *C. boninense* species complexes recovered from symptomatic plant tissues were tested for pathogenicity in different citrus species (two cultivars each of sweet orange, mandarin, lemon, lime, and grapefruit). Pathogenicity test was carried out following the standard techniques [40] on healthy detached leaves. Young leaves from two-year old plants were artificially inoculated with each isolate by placing three drops of aqueous suspension of 10^6^ spores per ml, or mycelia suspension. Spores were obtained from a week-old culture grown on PDA, suspended in sterile water, and filtered through two layers of sterile cheesecloth. In all tests, inoculation with sterile water was used as control. Each test isolate was inoculated on four leaves. Inoculated leaves were incubated on agar media for up to three weeks at 26°C. Inoculated leaves were assessed daily for the development of disease symptoms. At the end of each test, symptomatic tissues were surface disinfested and placed on water agar to confirm recovery of the inoculated isolates. Re-isolated cultures were examined for growth and morphological characteristics with the parent cultures. The experiment was conducted twice.

## Results

### PCR amplification

Three universal primer pairs (ITS-1F/ITS4, LROR/LR5 and ACT-512F/ACT-783R) were used to amplify the target ITS, LSU and ACT loci. All 167 fungal DNA samples were successfully amplified and sequenced. The amplified ITS, LSU and ACT loci were of approximately 600 bp, 900 bp and 300 bp in size, respectively. The average sizes of assembled sequences of the test isolates used in the present study were 570 bp for ITS, 855 bp for LSU, and 250 bp for ACT gene. Among isolates, there were only slight variations in amplicon size with few inconsistencies due to variable length nucleotide repeats.

### Identification of the test isolates

The multilocus sequences identified the associated fungi isolates to the species level. Among the 167 fungal isolates, 163 were identified as *C. gloeosporioides* (or its teleomorph *G. cingulata*) species complex (sensu lato; s.l.) using BLASTN search tool. Four isolates were recognized as *C. boninense* species complex.

The ITS sequence analysis delineated 163 isolates as *C. gloeosporioides* species complex (*C. aenigma=*2, *C. aotearoa*=2, and *C. gloeosporioides* sensu stricto [s.s.]=159). One isolate was resolved as *C. karstii*. Three isolates were identified as *C. truncatum*. The ITS sequence data resolved all the isolates to species level.

The analysis of portion of LSU revealed that 93 isolates were recognized as *C. gloeosporioides* s.l. (*C. asianum*=3, *C. fructicola*=4, and *C. gloeosporioides* s.s.=86), while four isolates were identified as *C. boninense* species complex. The rest 70 isolates were belonged to *G. cingulata*. The partial LSU data identified all the isolates to species level.

The partial ACT nucleotide sequence data resolved 163 isolates as *C. gloeosporioides* s.l. (*C. aenigma*=2, *C. asianum*=1, *C. crassipes*=1, *C. fructicola*=4, *C. siamense*=1, and *C. gloeosporioides* s.s.=154), one isolate as *C. karstii*, and three isolates as *C. magnisporum*. The partial ACT sequences discriminated all the isolates to species level.

The sequences of the ITS, LSU and ACT barcode markers discriminated all the *Colletotrichum* isolates studied to the species level. The results further indicated that CFLSD in Ethiopia have frequent association of *C. gloeosporioides* species complex.

### Phylogenetic analyses

**Figure 3:**
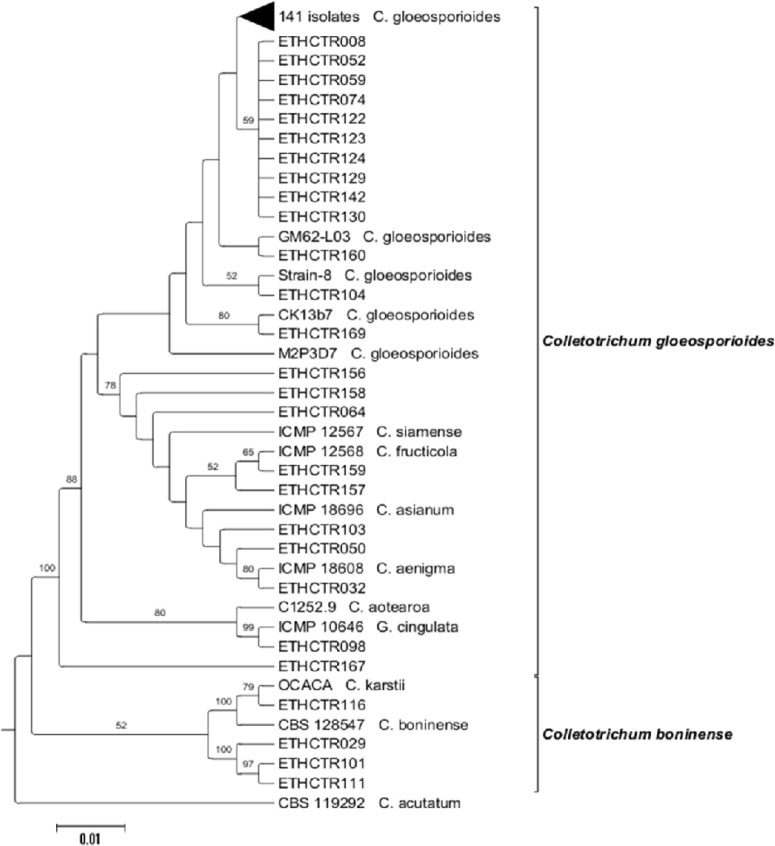
Neighbor-joining phylogenetic tree of concatenated ITS, LSU and ACT sequences of 167 Colletotrichum isolates generated in this study and 13 isolates from other studies, retrieved from the GenBank. Bootstrap support values (1000 replicates) of ≥ 50 are shown above the nodes. The tree was rooted with Colletotrichum acutatum (CBS 119292) as outgroup. The scale bar indicates the number of expected nucleotide changes per site.

**Figure 4:**
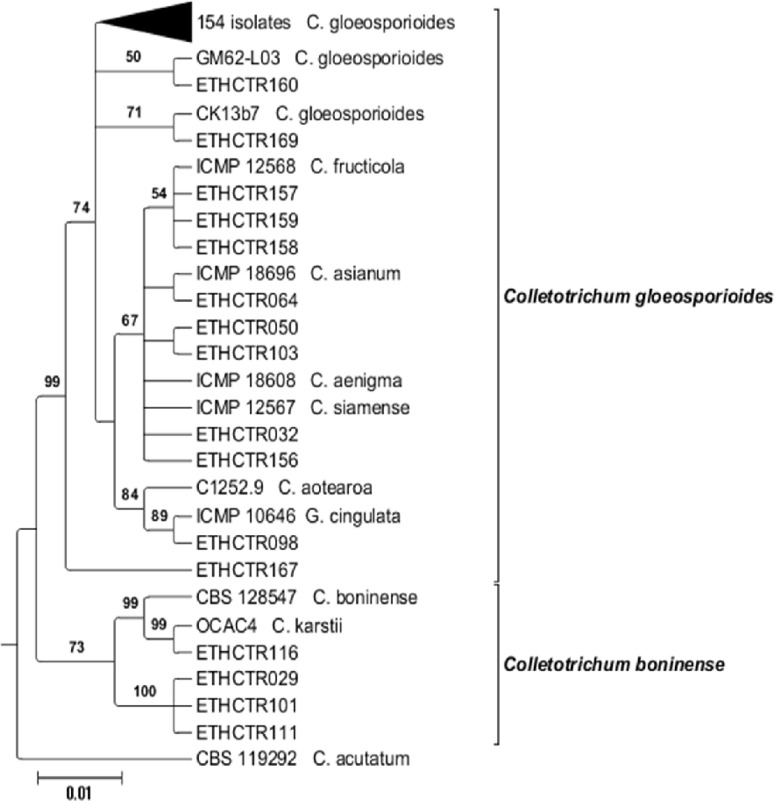
One of the most parsimonious trees obtained from a heuristic search of combined ITS, LSU and ACT sequences of 167 isolates generated in this study and 13 published reference isolates from the GenBank in the *Colletotrichum gloeosporioides* and *Colletotrichum boninense* species complexes. Bootstrap support values (1000 replicates) of ≥ 50 are shown at the nodes. *Colletotrichum acutatum* (CBS 119292) is used as outgroup. The scale bar indicates the number of expected changes per site.

**Figure 5:**
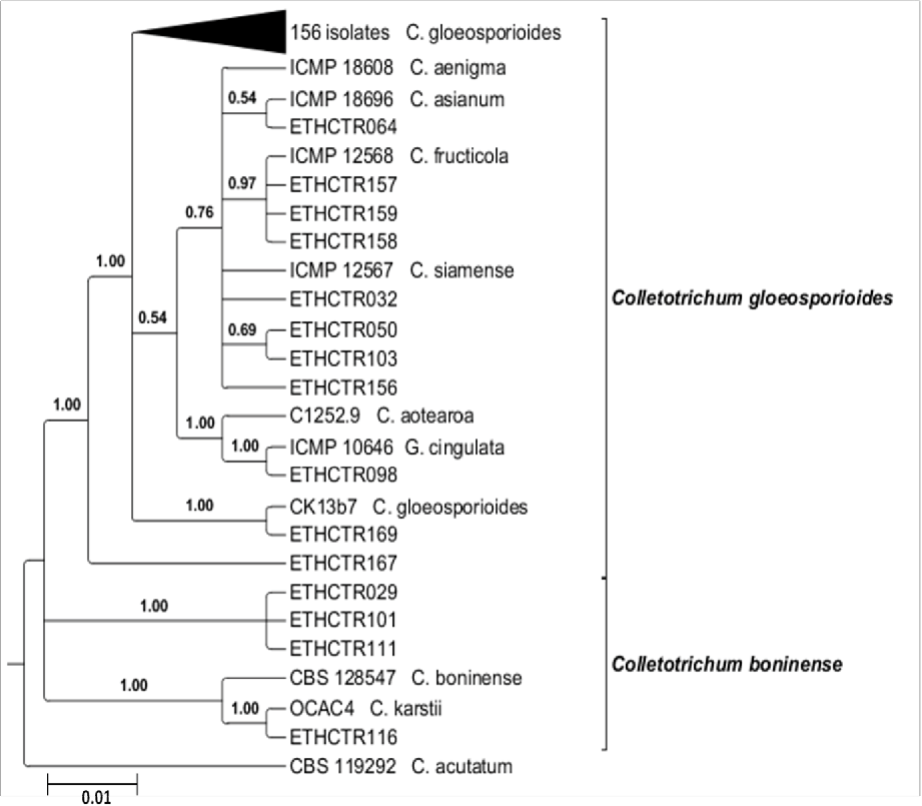
A Bayesian inference phylogenetic tree which illustrates the relationships of 167 isolates generated in this study and 13 published reference isolates from the GenBank in the *Colletotrichum gloeosporioides* and *Colletotrichum boninense* species complexes. The tree was built using concatenated sequences of the ITS, LSU, and LSU genes, each with a separate models of DNA evolution. Bayesian posterior probability values of ≥ 0.5 are shown above the nodes. *Colletotrichum acutatum* (CBS 119292) is used as outgroup. The scale bar indicates the number of expected changes per site.

The relatedness among fungal isolates was established through multi-gene phylogeny. The trees drawn from each individual dataset (ITS, LSU, and ACT loci) using NJ and MP had similar topology (data not shown) for the 70% reciprocal NJ bootstrap trees, which allowed us to combine them. The phylogenetic analysis of the combined sequences from the three loci using NJ, MP and Bayesian methods resulted similar grouping of isolates (Figures 3 to 5) into C. gloeosporioides [41] and C. boninense [42] species complexes.

In the combined multilocus analyses (gene boundaries of ITS: 1–527, LSU: 528-1385, ACT: 1386-1623) of 181 isolates including the 13 reference isolates and the outgroup (*C. acutatum* CBS 119292), 1913 characters including the alignment gaps were processed. Of these characters, 143 were parsimony informative; 236 were parsimony uninformative; and 1364 were constant. Parsimony analysis resulted in three most parsimonious trees. One of the most parsimonious trees (tree length=341, CI=0.584615, RI=0.829921, RC=0.485185, and HI=0.415385) obtained with the combined multiple sequence alignment of the three loci using MP method is presented in Figure 4.

The overall topology for all equally most parsimonious trees was similar; but they differed in the position of isolates within the clades. Out of the 8002 trees, 3001 trees were used to calculate the consensus tree and posterior probabilities. The analysis resulted in the delineation of four main clades within the isolates studied in this paper (Figure 5). Most of the isolates clustered in the first clade (*C. gloeosporioides* s.l.) with a bootstrap support and Bayesian posterior probability values of 74/1.0. The first main clade consists of several closely related species including *C. aenigma*, *C. asianum*, *C. fructicola* and *C. siamense* (67/0.76), *C. aotearoa* and *G. cingulata* (84/1.0), and *C. gloeosporioides* s.s. The second clade contained only one isolate, representing C. *gloeosporioides* s.l. (99/1.0). *Colletotrichum karstii* (99/1.0) and *C. boninense* (99/1.0) belonged to the third main clade. The fourth main clade consists of the *C. acutatum* isolate used as outgroup for the phylogenetic analysis [43-61].

### Pathogenicity tests

**Figure 6:**
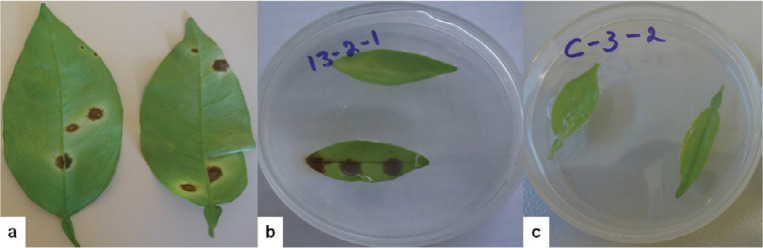
Pathogenic behaviour of the tested isolates of *Colletotrichum* on sweet orange leaves. (a) Naturally observed spots, (b) symptoms developed on artificially inoculated leaf, and (c) no symptom on water inoculated leaves.

Pathogenicity tests on detached leaves confirmed the ability of both *Colletotrichum* species complexes to produce the disease symptoms. Leaf spots symptom observed on citrus leaves under the field conditions are shown in Figure 6a. All test isolates in the *C. gloeosporioides* and *C. boninense* species complexes caused foliar disease symptoms on inoculated citrus leaves (Figure 6b), and the test isolates were consistently recovered from inoculated symptomatic leaf tissues. Some isolates caused necrosis of entire leaf area. Water inoculated controls remained healthy (Figure 6c).

## Discussion

Citrus fruit and leaf spot is a devastating disease of citrus in sub-Saharan Africa and Yemen [12]. A total of 167 fungal isolates were recovered from leaf and fruit samples with prominent symptoms of CFLSD. The multiple gene sequences distinguished all 167 isolates as members of genus *Colletotrichum* or teleomorph Glomerella which suggests that fruit and leaf spot disease of citrus in Ethiopia have complete association of *C. gloeosporioides* s.l. and *C. boninense* s.l. species complexes. The genus *Colletotrichum* is classified within the Fungi imperfecti. It belongs to the morphological classification of the phylum Ascomycota [51,62]. The fungus comprises *Colletotrichum* as anamorph or asexual state while *Glomerella* as sexual or teleomorph state [42,62]. Phylogenetic analysis of *Colletotrichum* reveals that the genus comprises nine major clades [62]. However, the taxonomy and phylogeny of *Colletotrichum* remains in a state of flux because many uncertainties exist with regard to the systematics of fungal pathogens from this genus [51]. The names *C. gloeosporioides* and *C. boninense* are commonly used in both broad (sensu lato) and strict (sensu stricto) senses. When used in a broad sense, they refer to the *C. gloeosporioides* [41] and *C. boninense* [42] species complexes.

*Pseudocercospora angolensis* has been reported as the causal agent of CFLSD [7]. In Ethiopia, Derso [6] and Yesuf [2] reported *P. angolensis* as the causal agent of CFLSD. Both authors tried to characterize the causal pathogen based on cultural and morphological characteristics. However, the pathogen description stated in both papers do not match with the typical characteristics of the *P. angolensis* reported by other scientists. Yesuf [2] indicated that apart from *P. angolensis*, *C. gloeosporioides* was found one of the important pathogens associated with citrus in the areas he surveyed. The infection of *P. angolensis* appears to predispose citrus fruits to secondary infection by *C. gloeosporioides* [10,16]. However, the isolations of *C. gloeosporioides* s.l. and *C. boninense* s.l. species complexes and no revival of *P. angolensis* from symptomatic citrus leaf and fruit samples in this study were highly surprising results. Such observations might have been attributed to the infection and colonization of host tissues by *Colletotrichum* species prior to sampling and/or the differences in the growth behavior between *Colletotrichum* and *Pseudocercospora* isolates. The former situation is more likely as all leaf and fruit samples had well developed disease symptoms at the time of collection. The latter situation may also hold true because of the relatively faster growth rate of *Colletotrichum* (6.3 mm/day) to *Pseudocercospora* (1.2 mm/day) [43,44] on general isolation medium which might have favored the frequent recovery of fast growing *Colletotrichum* species. Our efforts to isolate *P. angolensis* from leaves with early symptom of CFLSD also completed with isolation of *Colletotrichum* species at high frequency (Moges, Admassu, Belew, Yesuf, Maina and Ghimire unpublished results). The pathogenicity test of *C. gloeosporioides* s.l. and *C. boninense* s.l. isolates in detached leaf assay produced foliar symptoms and the test isolates were consistently recovered. These observations indicate important roles of *Colletotrichum* species on CFLSD. Therefore, inoculation study with *P. angolensis* and *Colletotrichum* species individually and in combination is important to determine the exact roles of these fungi in CFLSD initiation, development, and epidemics.

The *Colletotrichum* species complexes isolated in this study have been reported to show highly variable cultural and morphological characteristics [41,45], making these attributes less reliable to determine species complex [46]. Therefore, multiple gene based molecular phylogeny is preferred [47]. The multigene phylogeny adopted in this study discriminated 167 *Colletotrichum* isolates in two major groups: *C. gloeosporioides* and *C. boninense* species complexes with high species diversity (*C. aenigma*, C. *aotearoa*, *C. asianum*, *C. boninense* s.s., *C. crassipes*, *C. fructicola*, *C. gloeosporioides* s.s., *C. karstii*, *C. magnisporum*, *C. siamense*, *C. truncatum* and *Glomerella cingulata*). Although *C. acutatum* has been reported as the main pathogen of citrus anthracnose disease complexes worldwide [48], no isolate of *C. acutatum* was recorded from our study. Majority of taxa identified in this study have worldwide distribution, and many isolates cause diseases in agriculturally important crops [30,41,49,50]. Moreover, *Colletotrichum* is among ten most important plant pathogenic fungi [51].

*Colletotrichum gloeosporioides* species complex causes both preharvest diseases such as wither tip on twigs, tear stain [52] and fruit stem-end rot [53], and postharvest anthracnose [54] in various plant species. It is commonly associated with Key Lime Anthracnose and other postharvest diseases on citrus species [41]. It also causes post-bloom fruit drop on sweet orange in Brazil [48]. The ability of *C. fructicola* and *C. gloeosporioides* strains to cause anthracnose on citrus fruits has been demonstrated in China [55]. In the tropical Asia, *Colletotrichum siamense* causes anthracnose disease on a wide range of tropical fruits e.g. *Ficus racemosa*, *Azadirachta indica* and *Mangifera indica* [50]. Recently, *C. gloeosporioides* and *C. karstii* have been reported to cause severe lesions on sweet orange fruits (*Citrus sinensis*) in Italy, and *C. gloeosporioides* was more aggressive than *C. karstii* [56]. The virulence and pathogenicity of *C. asianum*, *C. fructicola*, and *C. karstii* have been demonstrated on various plant species including mango, papaya, banana, guava, and bell pepper [49].

*Colletotrichum boninense* s.l., once considered to belong to the *C. gloeosporioides* complex, was first described from *Crinum asiaticum* var. *sinicum* and *Cucumis melo* from Bonin Islands, Japan, where the species was associated with a variety of host plants [57]. Since then, this species has been reported as a pathogen causing leaf and fruit anthracnose on different host plants [42,45]. For instances, *C. boninense* has been found to be associated with diseases of Proteaceae in Australia and Zimbabwe, and with *Eucalyptus* in South Africa [58], *Dracaena* and *Pachira* in China, *Passiflora* in New Zealand and *Hippeastrum* in Brazil and the Netherlands [31], berries and twigs of *Coffea* in Vietnam [59], and avocado in Mexico [60]. *Colletotrichum* boninense and *C. gloeosporioides* have been shown to infect *Protea* leaves and stems in South Africa [61].

## Conclusion

The results of this study indicated a wider distribution of citrus fruit and leaf spot disease across citrus growing regions of Ethiopia. The multigene sequences (ITS, LSU, and ACT) analyses of all 167 fungal isolates recovered from CFLSD samples revealed them of genus *Colletotrichum* and surprisingly, none of the isolates were *Pseudocercospora angolensis*. Multigene phylogeny differentiated the 167 isolates into *C. gloeosporioides* s.l. or *C. boninense* s.l. species complexes. As of our knowledge, most of the taxa identified in this study are the first report on their association with citrus fruit and leaf spot symptoms in Ethiopia. The present findings suggest the absolute/ frequent association of *C. gloeosporioides* species complex with fruit and leaf spot symptoms of citrus in Ethiopia. We suggest the need for in-depth studies to examine roles of *P. angolensis*, *C. gloeosporioides* and *C. boninense* species complexes in citrus fruit and leaf spot disease epidemiology. This information would be helpful in developing effective disease management strategies.
